# Number of days required to measure sedentary time and physical activity using accelerometery in rheumatoid arthritis: a reliability study

**DOI:** 10.1007/s00296-023-05342-1

**Published:** 2023-05-25

**Authors:** Ciara M. O’Brien, George D. Kitas, Fiona Rayner, John D. Isaacs, Kenneth F. Baker, Arthur G. Pratt, Christopher D. Buckley, Karim Raza, Andrew Filer, Stefan Siebert, Iain McInnes, Andrew McGucken, Sally A. M. Fenton, The BIOFLARE Consortium

**Affiliations:** 1 School of Psychology, University of Surrey, Guildford, UK; 2 Department of Rheumatology, Russells Hall Hospital, Dudley Group NHS Foundation Trust, West Midlands, Dudley, UK; 3 Medical Research Council Versus Arthritis Centre for Musculoskeletal Ageing Research, University of Birmingham, Birmingham, UK; 4 Translational and Clinical Research Institute, Newcastle University, Newcastle Upon Tyne, UK; 5 Musculoskeletal Services Directorate, Newcastle Upon Tyne Hospitals NHS Foundation Trust, Newcastle, UK; 6 Kennedy Institute of Rheumatology, University of Oxford, Oxford, UK; 7 Rheumatology Research Group, Institute of Inflammation and Ageing, NIHR Birmingham Biomedical Research Unit, University of Birmingham, Birmingham, UK; 8 Research Into Inflammatory Arthritis Centre Versus Arthritis, College of Medical and Dental Sciences, University of Birmingham, Birmingham, UK; 9 Department of Rheumatology, Sandwell and West Birmingham NHS Trust, Birmingham, UK; 10 University Hospitals Birmingham NHS Foundation Trust, Birmingham, UK; 11 School of Infection and Immunity, University of Glasgow, Glasgow, UK; 12 School of Sport, Exercise and Rehabilitation Sciences, University of Birmingham, Edgbaston, Birmingham, B15 2TT UK

**Keywords:** Rheumatoid arthritis, Accelerometery, Sedentary behaviour, Sedentary time, Physical activity

## Abstract

This study aimed to determine the minimum number of days required to reliably estimate free-living sedentary time, light-intensity physical activity (LPA) and moderate-intensity physical activity (MPA) using accelerometer data in people with Rheumatoid Arthritis (RA), according to Disease Activity Score-28-C-reactive protein (DAS-28-CRP). Secondary analysis of two existing RA cohorts with controlled (cohort 1) and active (cohort 2) disease was undertaken. People with RA were classified as being in remission (DAS-28-CRP < 2.4, *n* = 9), or with low (DAS-28-CRP ≥ 2.4—≤ 3.2, *n* = 15), moderate (DAS-28-CRP > 3.2—≤ 5.1, *n* = 41) or high (DAS-28-CRP > 5.1, *n* = 16) disease activity. Participants wore an ActiGraph accelerometer on their right hip for 7 days during waking hours. Validated RA-specific cut-points were applied to accelerometer data to estimate free-living sedentary time, LPA and MPA (%/day). Single-day intraclass correlation coefficients (ICC) were calculated and used in the Spearman Brown prophecy formula to determine the number of monitoring days required to achieve measurement reliability (ICC ≥ 0.80) for each group. The remission group required ≥ 4 monitoring days to achieve an ICC ≥ 0.80 for sedentary time and LPA, with low, moderate and high disease activity groups requiring ≥ 3 monitoring days to reliably estimate these behaviours. The monitoring days required for MPA were more variable across disease activity groups (remission =  ≥ 3 days; low =  ≥ 2 days; moderate =  ≥ 3 days; high =  ≥ 5 days). We conclude at least 4 monitoring days will reliably estimate sedentary time and LPA in RA, across the whole spectrum of disease activity. However, to reliably estimate behaviours across the movement continuum (sedentary time, LPA, MPA), at least 5 monitoring days are required.

## Introduction

Device-based measures of free-living sedentary time and physical activity (PA) are increasingly used to investigate movement behaviours in clinical populations [1]. In studies of rheumatoid arthritis (RA), the hip-worn ActiGraph accelerometer (ActiGraph, Florida, USA) is commonly employed [2]. This device affords the ability to collect and store acceleration data (g), which is then processed using proprietary software (Actilife, ActiGraph, Florida, USA) to estimate daily sedentary time, light-intensity PA (LPA), moderate-intensity PA (MPA) and vigorous-intensity PA (VPA). However, daily patterns of movement behaviour show substantial intra-individual variation, which needs to be considered when estimating habitual patterns of activity from data collected over a relatively short time period.

Typically, participants are asked to wear an accelerometer for 7 consecutive days [3, 4]. However, research indicates adherence with 7-day monitoring protocols is low and can lead to reduced data quality [5, 6]. High participant burden is a common reason given for low adherence to 7-day accelerometer monitoring protocols. Investigating the minimum number of accelerometer monitoring days required to reliably estimate free-living sedentary time and PA will likely have important implications for increasing participant wear time and improving data quality.

Research has reported the minimum number of accelerometer monitoring days required to reliably estimate free-living sedentary time and PA vary in different populations [3]. Findings range from 4–9 days in children and adolescents, and 3–5 days in adults and older adults [7, 8]. No studies have examined the minimum number of accelerometer monitoring days required to reliably estimate free-living sedentary time, LPA and MPA in people with RA. This research is critical to inform researchers’ decision-making regarding protocol design (e.g., monitoring time frame) and accelerometer data reduction methods (e.g., minimum number of days needed) in studies utilising accelerometers to assess free-living behaviour in this patient group. Indeed, movement behaviours in people with RA are mechanically and physiologically unique from those of “healthy” adults, and intra-individual variation can be influenced by fluctuations in disease activity and rheumatic symptoms (e.g., pain and fatigue) [9, 10].

The aim of this study was therefore to determine the minimum number of ActiGraph accelerometer monitoring days required to reliably estimate free-living sedentary time and PA in people with RA. Given that RA is characterised by intermittent flares and fluctuations in disease activity, which could significantly impact free-living movement behaviours (and therefore reliability of accelerometer data), this study separately analysed data from two existing RA cohort studies with controlled (cohort 1) and active (cohort 2) disease, classified according to Disease Activity Score-28-C-reactive protein (DAS-28-CRP).

## Methods

### Participants (cohorts)

For cohort 1, data are taken from a study that aimed to identify BIOlogical Factors that Limit sustAined Remission in rheumatoid Arthritis (BIO-FLARE) [11]. Participants in this study were individuals diagnosed with RA [12, 13] with controlled disease/in remission (DAS-28-CRP < 2.4). Accelerometers were given to a sub-sample of participants in the BIO-FLARE study (*n* = 11), to understand the extent to which habitual levels of accelerometer-assessed sedentary time and PA are predictive of sustained remission vs. transition to active disease in people with RA. For cohort 2, data are taken from an observational longitudinal study that aimed to examine associations between accelerometer-assessed sedentary time and PA with RA outcomes [14]. Participants in this study were individuals diagnosed with RA [12] with low (DAS-28-CRP ≥ 2.4—≤ 3.2), moderate (DAS-28-CRP > 3.2—≤ 5.1) or high (DAS-28-CRP > 5.1) disease activity (*n* = 104).

The full protocols for the BIO-FLARE study (cohort 1) and observational longitudinal study (cohort 2) have been previously published, and detail the participant inclusion and exclusion criteria for the full studies [11, 14]. In both studies, inclusion criteria for accelerometer monitoring protocols were: aged ≥ 18 years, with the ability to ambulate independently (including with an assistive device). Wheelchair users and people who were pregnant were excluded from both studies. Written informed consent was obtained from all participants prior to undertaking any study procedures. These studies were approved by the North East–Newcastle and North Tyneside 1 Research Ethics Committee (cohort 1, 17/NE/0386, 26/02/2019 [ISRCTN registry identifier 16371380]), and the West Midlands National Health Service Research Ethics Committee (cohort 2, 16/WM/0371, 12/09/2016).

### Procedures

Procedures to characterise participants were identical across cohorts. Specifically, participants’ medical history, age, sex, ethnicity and date of diagnosis were recorded. Participants also undertook physical assessments including measurement of height (m) and weight (kg), and completed the Stanford Health Assessment Questionnaire (HAQ) to assess disease severity [15]. All participants also undertook routine clinical procedures to characterise their disease activity. Specifically, DAS-28-CRP was determined using; (1) a swollen-and-tender joint count in 28 joints (hands, wrists, elbows, shoulders, knees), (2) patient-reported degree of overall health (using a visual analogue scale) and (3) CRP (mg/L), which were entered into an online clinical DAS-28 calculator (https://www.das-score.nl/das28/DAScalculators/dasculators.html).

For assessment of free-living sedentary time, LPA and MPA, participants in cohort 1 wore an ActiGraph accelerometer for up to 6 months (to capture habitual PA prior to any flare occurring in the measurement period). For cohort 2, participants wore an ActiGraph accelerometer for 7 days. To standardise data across cohorts, the first 7 days of accelerometer data collection for each participant in cohort 1 was used in current analysis. In both cohorts, the device was attached via an elastic belt on the participants’ right hip [2]. Participants were asked to remove the accelerometer only for sleeping and water-based activities (e.g., bathing).

ActiGraph accelerometers record accelerations on the vertical (*Y*), horizontal right-left (*X*) and horizontal front-back (*Z*) axes [2]. Data on these axes are then used to calculate the vector magnitude (VM) using the equation, VM = √ (axis*Y*^2^ + axis*X*^2^ + axis*Z*^2^). Using Actilife, VM values are converted into “activity counts”, which are interpreted using researcher-selected accelerometer “cut-points” (thresholds) to determine the frequency, intensity and duration of free-living sedentary time and PA.

### Data reduction

Using Actilife, accelerometers were initialised to record data at a rate of 30 Hz and integrated into 1-s epochs upon download. Non-wear criteria were applied to device data (≥ 60 min of consecutive ‘0’ counts, with a spike tolerance of 2 min [16]) to identify “valid wear days” (i.e., accelerometer wear for ≥ 10 h/day). For participants’ data to be subsequently used in statistical analysis to determine measurement reliability, participants were required to have ≥ 6 valid wear days, including ≥ 1 weekend day [16, 17]. Estimates of daily sedentary time, LPA and MPA (min/day) were derived by applying recently validated RA-specific triaxial (VM) accelerometer cut-points [2]. To adjust for within and between participant variability in daily wear time, the proportion of daily time spent in these behaviours (%/day) were computed and used in analysis (e.g., day 1 sedentary time [%] = (day 1 sedentary time [min/day]/day 1 total wear time [min/day]) × 100).

### Statistical analysis

Statistical analysis was conducted using SPSS (v.24). Descriptive statistics were computed for time spent in sedentary behaviour, LPA and MPA (min/day and %/day), and the data distribution checked for normality using histograms and Q-Q plots.

One-way analysis of variance (ANOVA) was employed to examine within-participant differences in daily sedentary time, LPA and MPA (%/day) across days. Following this, single-day intraclass correlation coefficients (ICC) values were calculated (using a two-way random-effect model) to investigate the reliability of estimates (%/day) across the different days of accelerometer monitoring [8]. The single-day ICC values for sedentary time, LPA and MPA were entered into the Spearman-Brown Prophecy formula to establish how many days of accelerometer monitoring were required to reliably estimate time spent in sedentary behaviour, LPA and MPA in people with RA (N = ICCt/(1-ICCt) x (1-ICCs)/ICCs) [18]. Acceptable measurement reliability is achieved with an ICC ≥ 0.80 [3]. In the Spearman-Brown Prophecy formula, N = number of days required, ICCt = desired ICC value (0.80) and ICCs = single-day ICC value.

## Results

**Fig. 1:**
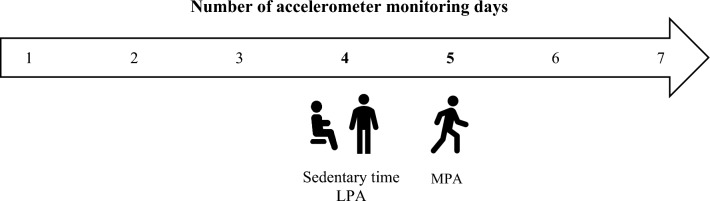
The number of accelerometer monitoring days recommended to measure movement behaviours across the spectrum of RA disease activity. *LPA* light-intensity physical activity, *MPA* moderate-intensity physical activity

Descriptive statistics are reported in Table 1. In cohort 1 (in remission, *n* = 11), *n* = 9 participants provided ≥ 6 valid days of data. In cohort 2 (*n* = 104), *n* = 72 participants provided ≥ 6 valid days of data (low DAS-28-CRP, *n* = 15; moderate DAS-28-CRP, *n* = 41; high DAS-28-CRP, *n* = 16). For both cohorts, one-way ANOVAs demonstrated no significant within-person differences in sedentary time, LPA and MPA estimates across days (*p* > 0.05). Single-day ICC values for sedentary time, LPA and MPA over the monitoring period (using %/day), as well as the required number of accelerometer monitoring days to achieve an ICC = 0.80, are displayed in Table 2. Figure 1 provides a visual representation of  results.

**Table 1:** Descriptive statistics for participants with controlled (cohort 1) and active (cohort 2) disease

	Cohort 1 Remission	Cohort 2 Low disease activity	Cohort 2 Moderate disease activity	Cohort 2 High disease activity
	(*n* = 9)	(*n* = 15)	(*n* = 41)	(*n* = 16)
Age (years)	65.1 (8.3)	62.3 (11.3)	62.7 (11.3)	55.1 (10.6)
Sex (*n* (%) female)	5 (56%)	9 (60%)	33 (81%)	9 (56%)
Ethnicity (*n* (%) Caucasian)	7 (78%)	14 (93%)	39 (95%)	15 (94%)
Physical characteristics				
Height (m)	1.7 (0.1)	1.6 (0.1)	1.6 (0.1)	1.6 (0.1)
Weight (kg)	75.5 (18.5)	72.1 (16.8)	77.3 (18.1)	94.7 (23.8)
BMI (kg/m^2^)	27.3 (6.5)	26.5 (3.8)	28.9 (5.3)	34.6 (6.8)
RA disease at enrolment				
RA duration (years)	7.2 (3.6)	11.7 (12.7)	13.2 (12.4)	9.3 (7.3)
Comorbidities (n)	1.9 (2.0)	1.1 (0.9)	1.1 (1.0)	2.7 (1.7)
DAS-28-CRP	1.6 (0.3)	2.9 (0.3)	4.1 (0.5)	6.0 (0.6)
HAQ	0.5 (0.9)	0.7 (0.7)	1.3 (0.6)	1.9 (0.5)
Accelerometer estimates				
Wear time (min/day)	858.9 (72.7)	913.3 (53.4)	889.2 (63.0)	871.8 (72.4)
Sedentary time (min/day)	651.3 (87.7)	709.1 (56.0)	689.3 (77.5)	705.2 (61.1)
LPA (min/day)	115.3 (26.1)	113.4 (35.6)	120.0 (37.8)	97.4 (35.7)
MPA (min/day)	92.3 (29.5)	90.8 (38.8)	79.9 (35.2)	69.1 (22.8)
Sedentary time (%/day)	75.6 (5.5)	77.7 (5.9)	77.5 (6.9)	81.1 (5.6)
LPA (%/day)	13.5 (3.4)	12.4 (3.7)	13.5 (4.3)	11.1 (3.8)
MPA (%/day)	10.8 (3.8)	9.9 (4.0)	8.9 (3.7)	7.9 (2.3)

^a^*n* number of participants, *BMI* body-mass index, *RA* rheumatoid arthritis, *DAS-28-CRP*, disease activity score-28, *C*-reactive protein, *HAQ* health assessment questionnaire, *LPA* light-intensity physical activity, *MPA* moderate-intensity physical activity. ^b^Values are mean (standard deviation) or *n* (percentage). ^c^Cohort 2 had missing data for height (*n* = 2), weight (*n* = 1), BMI (*n* = 2), RA duration (*n* = 4) and HAQ-DI (*n* = 1)

**Table 2:** Number of accelerometer monitoring days required to reliably estimate sedentary time and physical activity

	Day 1 (%)	Day 2 (%)	Day 3 (%)	Day 4 (%)	Day 5 (%)	Day 6 (%)	Single-day ICC	Number of days
Cohort 1 Remission	M (SD)	M (SD)	M (SD)	M (SD)	M (SD)	M (SD)	ICC value	N
Sedentary time	77.0 (7.4)	73.5 (6.9)	74.8 (7.6)	75.6 (5.7)	77.0 (8.5)	76.0 (5.7)	0.54	4
LPA	13.2 (4.8)	15.0 (4.3)	14.6 (4.4)	12.5 (4.1)	12.5 (5.0)	13.4 (3.8)	0.51	4
MPA	9.8 (5.1)	11.5 (3.6)	10.6 (4.6)	11.9 (5.9)	10.5 (5.4)	10.7 (3.2)	0.59	3
Cohort 2 Low disease activity	M (SD)	M (SD)	M (SD)	M (SD)	M (SD)	M (SD)	ICC value	N
Sedentary time	76.1 (6.0)	78.8 (6.3)	78.0 (7.1)	78.4 (7.8)	76.6 (7.6)	78.4 (6.3)	0.67	3
LPA	13.3 (3.4)	11.7 (3.6)	12.8 (5.6)	12.3 (4.8)	12.7 (4.2)	11.4 (3.6)	0.67	3
MPA	10.6 (4.0)	9.4 (4.7)	9.2 (4.2)	9.2 (4.3)	10.6 (5.0)	10.2 (4.3)	0.75	2
Cohort 2 Moderate disease activity	M (SD)	M (SD)	M (SD)	M (SD)	M (SD)	M (SD)	ICC value	N
Sedentary time	76.1 (8.2)	78.1 (8.1)	76.9 (7.6)	77.5 (8.6)	77.9 (8.2)	78.7 (8.3)	0.66	3
LPA	14.4 (4.9)	13.2 (4.8)	13.7 (4.7)	13.9 (5.2)	13.0 (5.0)	12.9 (5.1)	0.69	2
MPA	9.5 (4.6)	8.7 (4.1)	9.4 (5.1)	8.5 (4.3)	9.1 (4.6)	8.4 (4.2)	0.61	3
Cohort 2 High disease activity	M (SD)	M (SD)	M (SD)	M (SD)	M (SD)	M (SD)	ICC value	N
Sedentary time	80.6 (6.4)	82.6 (5.1)	82.2 (6.5)	80.2 (8.1)	80.5 (7.7)	80.3 (6.2)	0.61	3
LPA	11.0 (4.0)	10.5 (3.3)	10.4 (4.4)	11.5 (5.1)	11.7 (5.2)	11.2 (4.4)	0.67	2
MPA	8.4 (2.9)	6.9 (2.4)	7.4 (2.6)	8.3 (4.1)	7.7 (3.2)	8.4 (3.0)	0.49	5

^a^*ICC* intraclass correlation coefficient, *M* mean, *SD* standard deviation, *N* number, *LPA* light-intensity physical activity, *MPA* moderate-intensity physical activity

### Cohort 1

For participants in remission, single-day ICC values for sedentary time, LPA and MPA ranged between 0.51–0.59. To achieve an ICC ≥ 0.80, a minimum of 4 monitoring days would reliably estimate sedentary time and LPA, and a minimum of 3 monitoring days would be needed to reliably estimate MPA.

### Cohort 2

Single-day ICC values for sedentary time, LPA and MPA ranged between 0.67–0.75, 0.61–0.69 and 0.49–0.67 for participants with low, moderate and high disease activity, respectively. The minimum number of monitoring days required to achieve an ICC ≥ 0.80 were: low disease activity = 3 days (sedentary time, LPA) and 2 days (MPA); moderate disease activity = 3 days (sedentary time, MPA) and 2 days (LPA); high disease activity = 3 days (sedentary time), 2 days (LPA) and 5 days (MPA).

## Discussion

This is the first study to investigate the minimum number of accelerometer monitoring days needed to reliably estimate movement behaviours in RA. Results indicate that ≥ 4 monitoring days will reliably estimate sedentary time and LPA, and ≥ 5 monitoring days will reliably estimate MPA, across the whole spectrum of RA diease activity (i.e., in people with controlled or active disease). However, our results indicated measurement reliability may differ according to disease activity. Specifically, whilst ≥ 4 monitoring days produced acceptable ICC values for all movement behaviours among participants in remission, or with low or moderate disease activity, ≥ 5 monitoring days were required for participants with high disease activity. This is owing to the higher number of monitoring days required to measure MPA among those with DAS-28-CRP > 5.1.

Due to the lack of methodological accelerometer-based research in RA, “valid wear criteria” used in studies of healthy adults in the general population have largely informed the analytical decisions adopted in most existing RA studies (i.e., ≥ 10 h/day on ≥ 4 days, including ≥ 1 weekend day) [5, 6, 16]. Our finding that ≥ 4 monitoring days reliably estimates sedentary time and LPA in RA, aligns with these criteria, and may suggest that variability in lower-intensity movement behaviours is comparable between “healthy” adults and people with RA.

Whilst ≥ 4 monitoring days may be appropriate to reliably estimate sedentary time and LPA across the whole spectrum of RA disease activity, ≥ 5 monitoring days may be needed to measure MPA. Indeed, for those with high disease activity, current results indicate that ≥ 5 monitoring days is required to reliably estimate MPA. This suggests that relative to sedentary time and LPA, MPA is a less stable (more variable) behaviour in those with high disease activity, possibly due to the nature of RA disease flares and symptoms in this group. This reflects research that suggests disease activity and related rheumatic symptoms (e.g., pain and fatigue) are more strongly related to higher-intensity movement behaviours (i.e., MPA), relative to those at the lower end of the intensity spectrum. For example, Summers et al. [19] demonstrated a difference in accelerometer-assessed moderate-to-vigorous-intensity PA (MVPA) between individuals with “active disease” (mean DAS-28 = 5.3) and healthy controls, but no difference in accelerometer-assessed sedentary time between these groups. In addition, Haider et al. [20] showed that levels of accelerometer-assessed MVPA increased two-fold in individuals with a lower clinical disease burden (e.g., lower disease activity, pain intensity and functional disabilities). Further, a recent review highlighted pain, fatigue and compromised physical function as determinants of MVPA in RA, and suggested that such symptoms are less likely to hinder reductions in sedentary time and increases in overall PA (including LPA) in this patient group [21].

When we consider that most RA studies employ the aforementioned criteria of ≥ 4 monitoring days, the finding that ≥ 5 monitoring days are required to estimate MPA among individuals with high disease activity is problematic. Indeed, this analytical decision may not be appropriate in studies that focus on MPA, and where populations have more active disease (e.g., 19, 20). As such, the current study begins to address a methodological gap in the literature, and we recommend that both: 1) the movement behaviours of interest and 2) the disease activity status of the study sample, should be considered when making analytical decisions. Specifically, ≥ 4 monitoring days may be appropriate if studies are focused on sedentary time and/or LPA, or the population does not have high disease activity (e.g., average DAS28-CRP < 5.1). However, ≥ 5 monitoring days may be required to account for variability in MPA behaviours across days, among individuals with high disease activity.

Whilst this study provides a first step towards improving the rigour of protocols intended to measure movement behaviours in RA, there are some limitations to this study. First, this study examined measurement reliability in a hip-worn accelerometer protocol, following which activity count-based cut-points were applied to estimate sedentary time, LPA and MPA. Therefore, results may not be generalisable to wrist-worn accelerometer protocols, and where raw accelerometer data is analysed (vs. activity counts). Future research should be conducted using wrist-worn accelerometer protocols, and measurement reliability examined using raw accelerometer metrics (e.g., average acceleration, m*g*). The sample size of participants in remission was small (n = 9 with valid accelerometer data), and research with larger samples is required to confirm current findings.

Finally, our analysis did not account for other clinical factors that may have influenced variability in movement behaviours, such as comorbidities or disease severity. In this study, disease severity (assessed by the HAQ) was mild to moderate across both cohorts. However, the number of comorbidities was relatively higher in those with high disease activity (> 2 comorbidities, vs. > 1 in other groups). Indeed, the higher number of comorbidities may have contributed towards the higher number of monitoring days required to estimate MPA among those with high disease activity. As such, future work is needed to understand how such factors may impact measurement reliability in this patient group. With regard to disease severity specifically, the HAQ assesses physical function across several activities of daily living, including those which may not affect overall movement behaviours (e.g., gripping, reaching). Indicators of physical function that are more exclusively representative of whole body movement (e.g., rising, walking) may provide more insight into the impact disease severity and physical function has on the number of monitoring days required to reliably estimate sedentary time and PA in RA.

## Conclusion

This study provides the first evidence-based recommendations for valid accelerometer wear in people with RA, which considers variations in disease activity. We demonstrate that ≥ 4 monitoring days (each comprising ≥ 10 h/day) will reliably estimate sedentary time and LPA among people with RA, across the whole spectrum of disease activity. However, to reliably estimate behaviours across the movement continuum (sedentary time, LPA and MPA) ≥ 5 monitoring days are required.

## Data Availability

The data underlying this article will be shared on reasonable request to the corresponding author.

## Declarations

### Transparency statements

Part of this study’s protocol has previously been published (O’Brien et al., 2018). As a result, some of the text included herein may duplicate information reported in the prior publication.

## References

[1] Arvidsson D, Fridolfsson J, Borjesson M (2019). Measurement of physical activity in clinical practice using accelerometers. J Intern Med.

[2] O'Brien CM, Duda JL, Kitas GD, Veldhuijzen van Zanten JJCS, Metsios GS, Fenton SAM (2020). Measurement of sedentary time and physical activity in rheumatoid arthritis: an ActiGraph and activPAL validation study. Rheumatol Int.

[3] Matthews CE, Hagstromer M, Pober DM, Bowles HR (2012). Best practices for using physical activity monitors in population-based research. Med Sci Sports Exerc.

[4] Skender S, Ose J, Chang-Claude J, Paskow M, Brühmann B, Siegel EM (2016). Accelerometry and physical activity questionnaires a systematic review. BMC Public Health.

[5] Fenton SAM, Veldhuijzen van Zanten JJCS, Kitas GD, Duda JL, Rouse PC, Yu CA (2017). Sedentary behaviour is associated with increased long-term cardiovascular risk in patients with rheumatoid arthritis independently of moderate-to-vigorous physical activity. BMC Musculoskelet Disord.

[6] Fenton SAM, Veldhuijzen Van Zanten JJCS, Metsios GS, Rouse PC, Yu CA, Kitas GD (2018). Autonomy support, light physical activity and psychological well-being in Rheumatoid Arthritis: A cross-sectional study. Ment Health Phys Act.

[7] Trost SG, McIver KL, Pate RR (2005). Conducting accelerometer-based activity assessments in field-based research. Med Sci Sports Exerc.

[8] Sasaki JE, Junior JH, Meneguci J, Tribess S, Marocolo Junior M, Stabelini Neto A (2018). Number of days required for reliably estimating physical activity and sedentary behaviour from accelerometer data in older adults. J Sports Sci.

[9] Veldhuijzen van Zanten JJCS, Rouse PC, Hale ED, Ntoumanis N, Metsios GS, Duda JL (2015). Perceived barriers, facilitators and benefits for regular physical activity and exercise in patients with rheumatoid arthritis: a review of the literature. Sports Med.

[10] Thomsen T, Beyer N, Aadahl M, Hetland ML, Loppenthin K, Midtgaard J (2015). Sedentary behaviour in patients with rheumatoid arthritis: A qualitative study. Int J Qual Stud Health Well-being.

[11] Rayner F, Anderson AE, Baker KF, Buckley CD, Dyke B, Fenton S (2021). Biological Factors that limit sustained remission in rheumatoid arthritis (the BIO-FLARE study): protocol for a non-randomised longitudinal cohort study. BMC Rheumatol.

[12] Aletaha D, Neogi T, Silman AJ, Funovits J, Felson DT, Bingham CO (2010). 2010 Rheumatoid arthritis classification criteria: an American College of Rheumatology/European League Against Rheumatism collaborative initiative. Arthritis Rheum.

[13] Arnett FC, Edworthy SM, Bloch DA, McShane DJ, Fries JF, Cooper NS (1988). The American Rheumatism Association 1987 revised criteria for the classification of rheumatoid arthritis. Arthritis Rheum.

[14] O’Brien CM, Duda JL, Kitas GD, Veldhuijzen van Zanten JJCS, Metsios GS, Fenton SAM (2018). Correlates of sedentary behaviour and light physical activity in people living with rheumatoid arthritis: protocol for a longitudinal study. Mediterr J Rheumatol.

[15] Fries JF, Spitz P, Kraines RG, Holman HR (1980). Measurement of patient outcome in arthritis. Arthritis Rheum.

[16] Troiano RP, Berrigan D, Dodd KW, Masse LC, Tilert T, McDowell M (2008). Physical activity in the united states measured by accelerometer. Med Sci Sports Exerc.

[17] Semanik P, Song J, Chang RW, Manheim L, Ainsworth B, Dunlop D (2010). Assessing physical activity in persons with rheumatoid arthritis using accelerometry. Med Sci Sports Exerc.

[18] Eisinga R, Grotenhuis M, Pelzer B (2013). The reliability of a two-item scale: Pearson, Cronbach, or Spearman-Brown?. Int J Public Health.

[19] Summers G, Booth A, Brooke-Wavell K, Barami T, Clemes S (2019). Physical activity and sedentary behavior in women with rheumatoid arthritis: a comparison of patients with low and high disease activity and healthy controls. Open Access Rheumatol.

[20] Haider S, Sedlak M, Kapan A, Grabovac I, Lamprecht T, Erlacher L (2020). Factors associated with objectively measured physical activity in patients with seropositive rheumatoid arthritis. Int J Environ Res Public Health.

[21] Fenton SAM, O'Brien CM, Kitas GD, Duda JL, Veldhuijzen van Zanten JJCS, Metsios GS (2023). The behavioural epidemiology of sedentary behaviour in inflammatory arthritis: where are we, and where do we need to go?. Rheum Adv Pract.

